# Extensive T‐Wave Inversion Associated With Chest Pain: Elucidating the Underlying Truth

**DOI:** 10.1111/anec.70102

**Published:** 2025-06-30

**Authors:** Jing‐Xiu Li, Xin‐Xin Di, Min Gao, Xue‐Qi Li

**Affiliations:** ^1^ Department of Electrocardiogram The First Affiliated Hospital of USTC, Division of Life Sciences and Medicine, University of Science and Technology of China Hefei Anhui China; ^2^ Department of Cardiology The Fourth Affiliated Hospital of Harbin Medical University Harbin China

**Keywords:** catecholamine‐induced cardiomyopathy, pheochromocytoma, widespread T‐wave inversion

## Abstract

A patient with episodic chest pain, diaphoresis, amaurosis, and dizziness, along with a history of hypertension, presented with electrocardiographic findings of ST elevation in aVR, diffuse T‐wave inversion, and QTc prolongation. Initial diagnosis of NSTEMI was reconsidered after coronary angiography excluded significant stenosis, revealing myocardial bridging. Echocardiography and cardiac MRI showed preserved function without ischemia. Markedly elevated plasma renin and urinary normetanephrine, along with a retroperitoneal mass, suggested paraganglioma. Laparoscopic resection confirmed a 4.0 × 3.5 cm paraganglioma. This case highlights the importance of recognizing atypical ECG patterns that may mimic ischemia in catecholamine‐secreting tumors to guide timely diagnosis and intervention.

## Graphical abstract

Electrocardiographic manifestations, including ST‐segment elevation in lead aVR, extensive T‐wave inversion across leads I, II, III, aVF, and V_1_–V_6_, as well as prolongation of the corrected QT interval (QTc), are indicative of several clinical entities, such as acute coronary syndrome, myocarditis, pulmonary embolism, and Takotsubo cardiomyopathy secondary to ectopic pheochromocytoma.

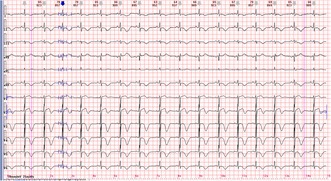

## 1 Case

**FIGURE 1:**
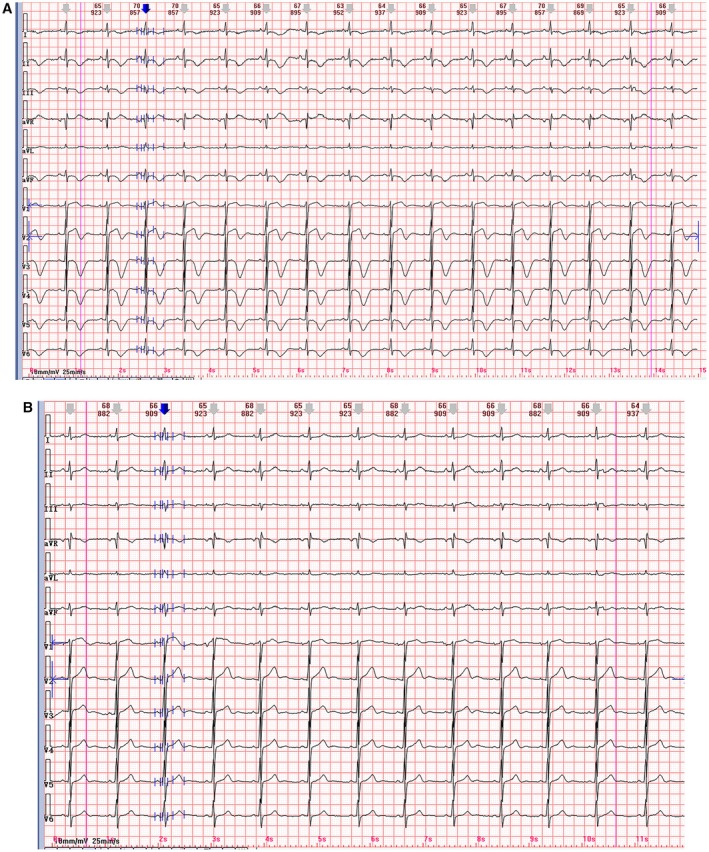
(A) An electrocardiogram exhibiting sinus rhythm with a ventricular rate of 67 beats per minute, characterized by ST‐segment elevation in lead aVR and widespread T‐wave inversion involving leads I, II, III, aVF, and V_1_–V_6_. Additionally, the corrected QT interval (QTc) was prolonged, measuring 463 ms. (B) Normalization of the ST‐T segments, QTc interval 430 ms, with a heart rate of 66 beats per minute.

A 38‐year‐old female patient presented to the cardiology department with a two‐week history of episodic chest pain, accompanied by diaphoresis, amaurosis, and dizziness. These symptoms were consistently alleviated upon rest. The patient reported a six‐month history of hypertension; the family medical history was unremarkable, with no known hereditary disorders identified. Throughout the clinical course, the patient's highest documented systolic blood pressure was 200 mmHg. The patient has been routinely treated with oral irbesartan‐hydrochlorothiazide, but blood pressure control remained suboptimal. Physical examination revealed a heart rate of 66 beats per minute (bpm), and blood pressure of 147/91 mmHg. Laboratory analyses revealed the following results: potassium was 3.79 mmol/L (reference range: 3.5–5.3 mmol/L); troponin I measured 0.15 ng/mL (reference range: 0–0.07 ng/mL); creatine kinase‐MB fraction was elevated at 53 IU (reference range: 0–25 IU); N‐terminal pro‐B‐type natriuretic peptide (NT‐proBNP) was significantly increased at 1455 pg/mL (reference range: 0–100 pg/mL); and D‐dimer concentration was within normal limits at 0.19 mg/L (reference range: 0.01–0.55 mg/L). Additionally, the white blood cell count (WBC) was 6.31 × 10^9^/L (reference range: 3.5–9.5 × 10^9^/L). An electrocardiogram (ECG) was conducted (Figure 1, panel A), and the resulting findings were evaluated by the Chief Cardiologist.

At presentation, the ECG (Figure 1, panel A) revealed sinus rhythm at a rate of 67 bpm, notable for ST‐segment elevation in lead aVR and widespread T‐wave inversion across leads I, II, III, aVF, and V_1_–V_6_. Additionally, the corrected QT interval (QTc) was measured at 463 ms. Following 20 days of comprehensive medical management, a repeat ECG (Figure 1, panel B) demonstrated resolution of the previously observed ST‐T segment abnormalities, a normalized QTc interval of 430 ms, and sinus rhythm at a rate of 66 bpm.

The provisional diagnosis was acute non‐ST‐segment elevation myocardial infarction (NSTEMI); however, coronary angiography excluded significant coronary artery stenosis but identified myocardial bridging in the mid‐left anterior descending artery. Echocardiography demonstrated preserved left ventricular function (ejection fraction: 65%), interventricular septal thickness of 13 mm, and posterior wall thickness of 11 mm. Cardiac magnetic resonance imaging showed no evidence of myocardial perfusion deficits or delayed enhancement abnormalities. Elevated plasma renin (66.24 pg/mL; approximately 2.0 times the upper reference limit of 32.8 pg/mL) and urinary normetanephrine (827.02 μg/g Cr; approximately 1.8 times the upper reference limit of 461 μg/g Cr), together with the identification of a hypervascular retroperitoneal mass, supported the suspicion of a paraganglioma. Surgical laparoscopic resection removed a solitary 4.0 × 3.5 cm mass near the abdominal aorta, with histopathology confirming paraganglioma (CgA+, S100+, Ki‐67: 20%, CK 8/18−). The patient was stable and discharged on postoperative day six.

## 2 Discussion

Pheochromocytoma is an uncommon, catecholamine‐secreting neoplasm associated with significant morbidity and mortality if unrecognized and untreated (Loscalzo et al. 2018; Zhang and Li 2021). Approximately 80% of catecholamine‐producing tumors originate in the adrenal medulla (referred to as pheochromocytomas), whereas the remaining 20% arise from extra‐adrenal sympathetic paraganglia and are termed paragangliomas. Of these extra‐adrenal paragangliomas, around 85% are located in the abdomen and pelvis, and approximately 15% in the thorax. These tumors originate from chromaffin cells located within the paraganglia of the autonomic nervous system, with adrenal tumors referred to as pheochromocytomas and extra‐adrenal counterparts as paragangliomas.

Electrocardiographic (ECG) abnormalities consistent with myocardial ischemia, injury, or left ventricular strain are often observed. The patient described herein presented with chest pain. Initial ECG (Figure 1, panel A) demonstrated sinus rhythm with ST‐segment elevation in lead aVR, extensive T‐wave inversions in leads I, II, III, aVF, and V_1_–V_6_, accompanied by prolongation of the corrected QT (QTc) interval. These findings, along with mildly elevated serum troponin I levels, initially suggested acute NSTEMI, raising concern for possible left main coronary artery or multivessel coronary artery disease. However, subsequent coronary angiography excluded coronary artery disease, revealing normal left main coronary artery anatomy without stenosis. Myocardial bridging was identified in the mid‐segment of the left anterior descending artery, whereas the left circumflex and right coronary arteries exhibited no significant stenosis, thus eliminating NSTEMI as a diagnosis.

Further evaluation via contrast‐enhanced cardiac magnetic resonance imaging (CMR) demonstrated neither significant perfusion deficits nor delayed myocardial enhancement in the left ventricle, indicative of an absence of myocardial infarction or inflammation. Additionally, normal white blood cell counts and absence of recent respiratory infection excluded acute myocarditis. QT interval prolongation with simultaneous T‐wave inversion in anterior and inferior ECG leads can suggest acute pulmonary embolism (PE) (Zhao et al. 2015). Although the patient exhibited similar ECG abnormalities, normal D‐dimer values effectively excluded acute PE. Given the patient's age and sex, Takotsubo (stress) cardiomyopathy was considered in the differential diagnosis. However, the absence of regional wall motion abnormalities on echocardiography, lack of apical ballooning on cardiac MRI, and no preceding emotional or physical stressor rendered this diagnosis unlikely. Given the markedly elevated plasma renin and urinary normetanephrines in the context of persistent hypertension, a contrast‐enhanced CT urography (CTU) was performed, which identified a hypervascular mass in the right retroperitoneal space, raising suspicion for a paraganglioma.

Urinary normetanephrine was elevated at 827.02 μg/g Cr (approximately 1.8 times the upper reference limit), a level that falls within the equivocal range described in prior studies. However, when considered alongside markedly elevated plasma renin and a hypervascular retroperitoneal mass, this finding supported the diagnosis of sympathetic paraganglioma. Surgical excision via laparoscopy removed a solitary 4.0 × 3.5 cm mass adjacent to the abdominal aorta. Immunohistochemical analysis confirmed paraganglioma (CgA+, S100+, Ki‐67 proliferation index 20%, CK8/18−). Postoperative follow‐up ECG revealed normalization of ST‐T segments, QTc interval shortening to 430 ms, and restoration of previously inverted T‐waves. Given the absence of familial sudden cardiac death history and observed QTc interval normalization, long QT syndrome was excluded. Echocardiography indicated preserved left ventricular systolic function (ejection fraction: 65%), mild interventricular septal (13 mm) and posterior wall thickening (11 mm). Although initial ECG alterations were suggestive of hypertensive cardiomyopathy, postoperative normalization indicated catecholamine‐induced cardiomyopathy, specifically Takotsubo cardiomyopathy secondary to paraganglioma. Although the patient had been chronically treated with irbesartan and hydrochlorothiazide, dedicated alpha‐adrenergic blockade was not initiated preoperatively, as the diagnosis of paraganglioma was only made after CTU imaging. Despite this, the patient remained hemodynamically stable during laparoscopic excision, likely due to the moderate tumor size and lack of overt catecholamine crises. This case underscores the importance of early recognition and biochemical confirmation to enable optimized preoperative pharmacologic preparation when catecholamine‐secreting tumors are suspected. Clinicians must recognize these distinctive ECG features to facilitate timely diagnosis and appropriate management. According to current WHO guidelines, all paragangliomas should be considered tumors of uncertain malignant potential, regardless of histological appearance. In our case, the Ki‐67 index was 20%, suggestive of an aggressive phenotype. Therefore, the patient was advised to undergo long‐term surveillance with regular biochemical monitoring and periodic imaging to detect any recurrence or metastatic progression. This aligns with contemporary recommendations for postoperative follow‐up in patients with paraganglioma.

In young patients with severe or refractory hypertension, episodic chest pain, and ischemic ECG changes—unexplained by coronary, myocardial, or pulmonary pathology—paraganglioma should be considered. Early biochemical testing with plasma or urinary metanephrines, or timely contrast‐enhanced abdominopelvic imaging, is critical for accurate and prompt diagnosis.

## Author Contributions

Jing‐Xiu Li and Xue‐Qi Li contributed significantly to data collection and manuscript preparation. Xin‐Xin Di and Min Gao performed the analysis with discussion. All authors agree on the order in which their names will be listed in the manuscript.

## Ethics Statement

We identify that the ethics committee of The First Affiliated Hospital of USTC has approved the case and that this case conforms to recognized standards, the Declaration of Helsinki.

## Conflicts of Interest

The authors declare no conflicts of interest.

## Data Availability

The data that support the findings of this study are available from the corresponding author upon reasonable request.
